# Association of genetic variants in chromosome 17q21 and adult-onset asthma in a Chinese Han population

**DOI:** 10.1186/1471-2350-12-133

**Published:** 2011-10-11

**Authors:** QiuRong Fang, Hailing Zhao, Aihua Wang, Yaoqin Gong, Qiji Liu

**Affiliations:** 1 Key Laboratory for Experimental Teratology of the Ministry of Education and Department of Medical Genetics, Shandong University School of Medicine, Jinan, Shandong 250012, PR China; 2 Department of Respiratory Internal Medicine, Qilu Hospital, Jinan, Shandong 250012, PR China

## Abstract

**Background:**

Genome-wide association studies of asthma have identified a novel region containing *ORMDL3 *at chromosome 17q21 that is strongly associated with childhood-onset asthma and significantly linked to *ORMDL3 *transcript abundance. These results have been successfully replicated in childhood-onset asthma cohorts in several ethnic groups. In this study, we aimed to evaluate the association of polymorphisms in *ORMDL3, GSDMB, ZPBP2 *and *IKZF3 *and adult-onset asthma in a Chinese Han population.

**Methods:**

We genotyped 5 single nucleotide polymorphisms (SNPs) at chromosome 17q21 in 1,366 Han Chinese people comprising 710 patients with adult-onset asthma and 656 healthy controls. We compared the 2 groups in terms of allele and haplotype frequencies. Transcript levels were measured in leukocytes from 61 asthma patients by quantitative real-time PCR.

**Results:**

We found the 5 SNPs significantly associated with asthma (P<0.05), of which 2, rs11557467 and rs9303277, were strongly associated (P<0.001). Subjects carrying the G allele of rs11557467 or the C allele of rs9303277 showed increased risk of asthma (odds ratio [OR] 1.27, 95% confidence interval 1.07-1.51, P = 0.006, and OR 1.27, 1.07-1.49, P = 0.005, respectively), even after adjusting for age and sex. The risk of asthma was lower for carriers of the haplotype CTGTT (OR 0.81, 0.67-0.97, P = 0.02). The risk allele for each SNP was associated with increased expression of *ORMDL3 *and *GSDMB *in leukocytes (all p<0.05).

**Conclusions:**

Our replication study suggests that variants in 17q21 are significantly associated with risk of adult-onset asthma and gene expression in a Chinese Han population.

## Background

Asthma is a complex disease triggered by the interaction of genetic predisposition and environmental factors [1,2]. Recently, the first genome-wide association study (GWAS) of asthma in European subjects identified a novel region containing the *ORM1-like 3 *(*ORMDL3*) gene at chromosome 17q21 strongly associated with childhood asthma and *ORMDL3 *transcript abundance [3].

*ORMDL3*, located at 17q21.1, belongs to a novel gene family that also includes *ORMDL1 *and *ORMDL2*[4]. Breslow et al. recently found Orm proteins as mediators of sphingolipid homeostasis and suggested that sphingolipid misregulation might be related to the development of childhood asthma [5]. *GSDMB*, encoding the protein gasdermin B, a member of the cancer-associated gasdermin-domain-containing protein family implicated in cancer pathogenesis [6], is adjacent to *ORMDL3 *on chromosome 17.

*ORMDL3 *as a candidate gene for asthma susceptibility has been replicated in 8 white populations (Scottish [7], French-Canadian [8,9], French [8], German and British ancestry [3], North Americans of European ancestry [10], Mexicans in Mexico City [11] and Australia [12]), 3 ethnically diverse populations (Mexicans, Puerto Ricans and African Americans) [13], and 2 Asian populations (Japanese [14] and Chinese [Hong Kong][15]). However, all these associations were for childhood asthma.

To validate whether *ORMDL3 *is a risk factor for adult-onset asthma, we sought to replicate the association of polymorphisms in *ORMDL3, GSDMB, ZPBP2 *and *IKZF3 *and asthma in an adult Chinese Han population. We also determined whether these variants are associated with gene levels in leukocytes.

## Methods

### Subjects

We included 1,366 unrelated subjects of exclusively Han ethnicity in this case-control study. From 2008 to 2009, we recruited 710 adult-onset asthma patients from Qilu Hospital, Jinan. All patients met the 1987 criteria for asthma of the American Thoracic Society (ATS, 1987). We also recruited 656 unrelated, random-sampled healthy controls who were matched to cases by age, ethnicity and geographic region, who underwent comprehensive medical screening at Qilu Hospital and were without symptoms or history of asthma or other pulmonary diseases or atopy. Healthy controls had no first-degree relatives with asthma nor history of asthma or atopy at the time of recruitment [16].

The mean age of asthma onset in patients was 25 years. Onset age was confirmed by physicians asking the question "How old were you when you first experienced the asthma symptoms of wheezing, shortness of breath, chest tightness and coughing?" Patients with vague answers to the question were not included. We included only patients with asthma onset at age 18 years or older. Clinical and demographic characteristics of patients and controls are in Table 1.

**Table 1:** Clinical and demographic characteristics of patients and controls

Characteristics	Patients	Controls
No. of subjects	710	656
Age, mean (range), years	28.7 (22-37)	29.1 (20-42)
Sex (male/female)	345/365	310/346
FVC (% predicted), mean±SD	67.7±17.2	80.6±15.4
FEV1 (% predicted), mean±SD	56.4±16.8	87.9±12.2
% change in FEV1 by bronchodilator, mean±SD	28.2±14.6	4.3±3.9

FVC: forced vital capacity; FEV_1_: forced expiratory volume in 1 s.

Blood samples were obtained from all participants after their informed consent. Genomic DNA was extracted from peripheral blood leukocytes by a standard phenol-chloroform method. Total RNA was extracted from leukocytes by the TRIZOL reagent method (Invitrogen, Carlsbad, CA). For transcript analysis, we selected samples for 61 patients who were first-episode asthma patients and had not received any medication within the previous month.

The study was approved by the ethics review committee for human studies at the School of Medicine, Shandong University.

### Single nucleotide polymorphism selection and genotyping

We selected 5 single nucleotide polymorphisms (SNPs) according to previous results and the linkage disequilibrium (LD) status in HCB. rs7216389, rs12603332 and rs12936231 were genotyped by PCR-restriction fragment-length polymorphism analysis. PCR amplification conditions were optimized with an initial denaturation step at 95°C for 5 min, then 35 cycles of denaturation at 94°C for 40 s, annealing at optimal temperature for 40 s and extension at 72°C for 50 s, and a final extension at 72°C for 10 min in a thermal cycler. Then, PCR products were digested with locus-specific restriction endonucleases (New England BioLabs, Beijing) and were electrophoresed on agarose gels. rs9303277 and rs11557467 were genotyped by the TaqMan SNP genotyping method with assay-on-demand probes and primers (C_9272050_20 for rs9303277, C_9272244_10 for rs11557467, Applied Biosystems, Foster City, USA). Genotyping accuracy was confirmed by direct sequencing of PCR products for 5% randomly chosen samples. Primers and restriction endonucleases are in Table 2.

**Table 2:** Primers used in PCR-restriction fragment-length polymorphism (RFLP) and gene splicing analysis

SNP	alleles	PCR primer	PCR product	Restriction enzyme	RFLP products (bp)
rs7216389	T/G	F:5'-GTCACATTTCCACCAGTT-3' R:5'-CTGTAATCCCAGCACTTT-3'	563bp	Nsi I	334/229
rs12603332	A/C	F:5'-GAGTGTCTGGCATACTGGCTGG-3' R:5'CCGAAAACTTCTGCTGCCATAGCTGGCACG-3'	214bp	BstU I	174/30
rs12936231	C/G	F:5'-TATGACATATTGTTGCTTCT-3' R:5'-ATAGACTCACAAAGGGATTC-3'	349bp	Bsl I	191/158
rs12603332 (gene splicing)		F1:5'-GACCCTCACCAACCTCATTCAC-3' R1:5'-CCATAATCCATCTGCTCCCAGTG-3'	129bp		
		F2:5'- AGGGAATGGGAAGGGCTCAC-3' R2:5'- CGGCATGTGGCTGACAAGTG-3'	801bp		

### Gene splicing

The SNPs located in the intron may affect mRNA splicing. According to the ensemble database, *ORMDL3 *contains 2 transcripts: ENST00000304046 (ORMDL3-001) and ENST00000394169 (ORMDL3-002). To assess whether rs12603332, located in the first intron, might affect gene splicing, we designed 2 pairs of primers. One pair, located in the first and second exons, would amplify both transcripts. Another pair, located in the 3' untranslated region of the second transcript, would yield products only when the second transcript was present. We conducted this experiment in 10 samples, half of them containing the CC genotype and half the TT genotype in the rs12603332 site. Primer sequences are available upon request.

### Transcript level analysis

To determine the impact of rs7216389, rs12603332, and rs12936231 SNPs on the gene expression of *GSDMB *and *ORMDL3*, we performed real-time PCR for different genotype groups using peripheral leukocyte RNA from 61 patients. We determined relative mRNA levels of human *ORMDL3*, and *GSDMB*, with human *GAPDH *mRNA used as an internal control, using an ABI 7500 real-time PCR system. The comparative threshold cycle method (C_T_) was used to quantitate mRNA. Four duplicate wells were used for each subject.

### Statistical analysis

SNPs were tested for deviation from Hardy-Weinberg equilibrium by chi-square test. Categorical data were compared by Fisher's exact test and allelic expression level by one-way ANOVA. The allele and genotype frequencies, odds ratios (ORs) with 95% confidence intervals (95% CIs), and p values were analyzed by use of PLINK 1.07[17]. Pairwise LD was measured by use of Haploview 4.2. Five-locus haplotypes were estimated with a full-precise-iteration (FPI) algorithm by use of the online SHEsis software[18]. A p < 0.05 was considered statistically significant.

## Results

### Association study

Genotype and allele frequencies for each SNP are in Table 3. The distributions of the 5 SNPs were in Hardy-Weinberg equilibrium (P>0.05) for both patients and controls. To test the association of each locus with asthma, we compared cases and controls in terms of allele frequency and genotype distribution for each polymorphism. All SNPs were significantly associated with asthma, even after adjusting for age and sex.

**Table 3:** Genotype and allele association analysis of 5 single nucleotide polymorphisms

SNP ID	Polymorphisms Genotype/allele	Patients with asthma, n (%)	Controls, n (%)	OR	95% CI	*P *value	P*
rs7216389 (*GSDMB*)	TT	384 (55.2)	298 (46.8)	1			
	CT	258 (37.1)	286 (44.9)	1.43	1.14-1.80	0.008	
	CC	54 (7.7)	53 (8.3)	1.27	0.84-1.90		0.01
	T	1026 (73.7)	882 (69.2)	1		0.011	
	C	366 (26.3)	392 (30.8)	1.25	1.05-1.48		
							
rs12603332 (*ORMDL3*)	CC	385 (54.2)	302 (46.04)	1			
	CT	268 (37.8)	300 (45.73)	1.43	1.14-1.784	0.007	
	TT	57 (8.0)	54 (8.23)	1.21	0.81-1.81		0.01
	C	1038 (73.1)	904 (68.9)	1		0.016	
	T	382 (26.9)	408 (31.1)	1.23	1.04-1.45		
							
rs12936231 (*ZPBP2*)	CC	393 (56.1)	298 (47.8)	1			
	CG	257 (36.7)	278 (44.6)	1.43	1.14-1.79	0.009	
	GG	50 (7.1)	47 (7.5)	1.24	0.81-1.90		0.01
	C	1043 (74.5)	874 (70.0)	1		0.013	
	G	357 (25.5)	372 (30.0)	1.24	1.05-1.48		
							
rs11557467 *(ZPBP2)*	GG	383 (55.51)	300 (46.80)	1			
	GT	257 (37.25)	288 (44.93)	1.43	1.14-1.79	0.006	
	TT	50 (7.25)	53 (8.27)	1.35	0.89-2.05		0.005
	G	1023 (74.13)	888 (69.27)	1		0.006	
	T	357 (25.87)	394 (30.73)	1.27	1.07-1.51		
							
rs9303277 *(IKZF3)*	CC	348 (50.58)	269 (42.43)	1			
	CT	278 (40.41)	295 (46.53)	1.37	1.09-1.73	0.012	
	TT	62 (9.01)	70 (11.04)	1.46	1.00-2.13		0.004
	C	974 (70.78)	833 (65.69)	1		0.005	
	T	402 (29.22)	435 (34.31)	1.27	1.07-1.49		

SNP, single nucleotide polymorphism; OR, odds ratio; 95% CI, 95% confidence interval; P* adjusted for age and sex

### Gene splicing

**Figure 1:**
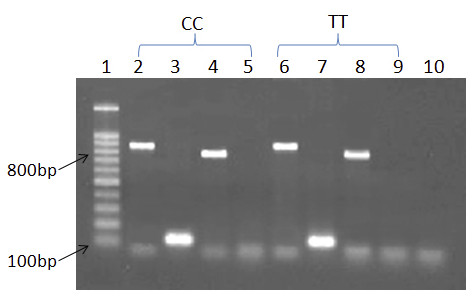
**Absence of alternate exon splicing in ORMDL3**. Subjects with the CC genotype are in lanes 2 to 5 and those with the TT genotype are in lanes 6 to 9. We used cDNA as a template in lanes 3, 5, 7, and 9 and genomic DNA as a template in lanes 2, 4, 6, and 8. The first pair of primers was used in lanes 2, 3, 6, and 7 and the second pair of primers in lanes 4, 5, 8, and 9. However, only the first pair of primers worked in cDNA for subjects with both CC and TT genotypes. Lanes 1 and 10 are the 100-bp markers and PCR negative control, respectively.

PCR results were similar for both CC and TT homozygotes at rs12603332 (Figure 1). Only the first pair of primers yielded products for all cDNA samples. However, the second pair of primers yielded PCR products when genomic DNA was used as a template. Therefore, only the ORMDL3-001 transcript was expressed in all samples.

### Transcript level analysis

**Figure 2:**
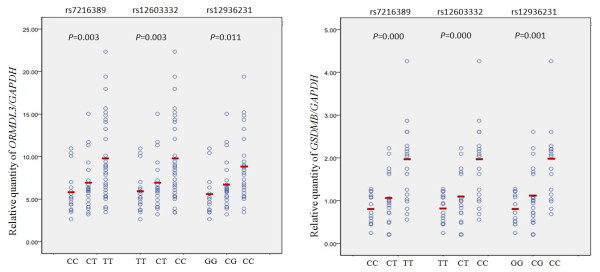
**Expression of *ORMDL3 *and *GSDMB *by genotype**. The transcript levels of target genes in each sample were normalized to that of GAPDH. Each dot represents the mean value of 4 replicates for each subject. The mean value for each group is indicated with red bars.

The SNPs rs7216389, rs12603332, and rs12936231 had striking effects on *ORMDL3 *and *GSDMB *expression (Figure 2). The expression level was robustly increased in homozygotes for risk alleles as compared with those carrying 1 or 2 copies of the reference alleles.

### LD evaluation and haplotype analysis

**Figure 3:**
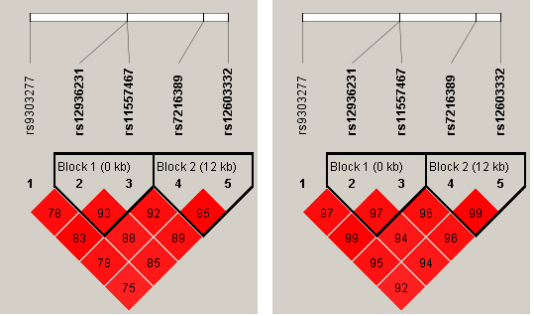
**Linkage disequilibrium of the 5 single nucleotide polymorphisms**. Left is r^2 ^and right is D'.

As expected, all 5 SNPs were in strong LD, with r^2 ^> 0.75 (Figure 3). This result is consistent with the data from Hapmap HCB. We identified 11 different haplotypes by haplotype analysis (Table 4). However, only 3 had a frequency > 1%. The haplotype analysis results were the same as the single-site association results. As compared with the haplotype TCCCG, CTGTT was a risk factor, with OR = 0.81 (95% CI 0.67-0.97, p 0.021).

**Table 4:** Haplotype frequencies of the 5 single nucleotide polymorphisms in cases and controls.

Haplotype	Cases, n (%)	Controls, n (%)	P	OR (95% CI)
T C C C G	903 (0.681)	735 (0.650)	Ref	1.00
T C C T G	45 (0.034)	39 (0.035)	0.968	1.06 (0.69-1.65)
C T G T T	324 (0.244)	327 (0.289)	0.021	0.81 (0.67-0.97)

The order of 5 SNPs: rs7216389, rs12603332, rs12936231, rs9303277, rs11557467 95% CI, 95% confidence interval; OR, odds ratio; Ref, reference haplotype used for comparison.

## Discussion

Family and twin studies have implicated genetic components in the pathogenesis of asthma [19,20]. However, although several asthma susceptibility genes have been identified, the complex etiology, combined with extensive heterogeneity, has made genetic studies of asthma challenging. Recently, the first GWAS for asthma identified ORMDL3 as a new asthma candidate gene[3], and subsequent studies confirmed the association of polymorphisms in the chromosome region with asthma in different ethnic groups[3,7-11,13-15]. In 2010, Moffatt et al. confirmed ORMDL3 as an important asthma susceptibility gene in their consortium-based GWAS[21]. In this study, we confirmed the association of chromosome 17q21 SNPs and expression of asthma-associated genes with susceptibility to asthma in an adult Chinese Han population.

Our results are different from many previous studies finding an association of 17q21 variants and childhood-onset asthma. Our study is among the first to report the association of genetic variants in this locus and adult-onset asthma. Not much is known about the differences between early- and adult-onset asthma in terms of disease mechanisms. We believe childhood- and adult-onset asthma may share certain disease mechanisms. Exposure to strong or cumulative environmental triggers such as pollution or infection may be responsible for adult-onset cases. Such triggers may not have existed during the patients' childhood or might have been weaker, such as for patients living in regions under rapid transition from agricultural economy to industrialization. Our results, from a genetics viewpoint, supports the notion that similar mechanisms may exist in childhood- and adult-onset asthma.

Our study contains some limitations in that it may reflect recall bias in self-reported age of asthma onset. Also, the 5 SNPs studied were in complete LD, so we did not perform a multiple test correction to avoid increasing the II error.

Although GWASs have successfully identified common variants associated with a wide variety of complex diseases, the design cannot establish causality of disease-associated SNPs. For the variants at chromosome 17q21, which are in a large strongly LD region, disentangling which gene or SNP is the true functional locus that contributes independently to the asthma susceptibility is difficult.

Many groups have tried to identify causal variants. Recently, Verlaan et al. discovered a striking correlation between rs12936231 and *ORMDL3, GSDMB *and *ZPBP2 *gene expression, and this variant may be one of the causal SNPs because it alters the binding of insulator protein CTCF (CCTC binding protein)[22]. Gerard Cantero-Recasens et al. revealed that *ORMDL3 *could inhibit the expression of the sarco-endoplasmic reticulum Ca^2+ ^pump and lead to reduced endoplasmic reticulum Ca^2+ ^concentration and increased unfolded-protein response[23], which suggests that *ORMDL3 *might be involved in inflammation and asthma.

## Conclusions

In summary, our case-control study revealed the 5 SNPs we studied significantly associated with asthma in an adult Han Chinese popoulation, but no SNP had an independent effect. In agreement with previous studies, variants rs7216389, rs12603332 and rs12936231 had striking effects on *ORMDL3 *and *GSDMB *gene expression, with the presence of the risk allele for each SNP robustly increasing the expression of the genes. Further studies are required to elucidate the mechanism of 17q21 variants predisposing asthma susceptibility.

## Competing interests

The authors declare that they have no competing interests.

## Authors' contributions

QL conceived and designed the study. RQ conducted the laboratory experiments, developed protocols, performed the statistical analysis, and drafted the manuscript, HZ helped with genotyping, and YG and QL polished the final manuscript. AW participated in the clinical survey and sample collections. All authors read and approved the final manuscript.

## Pre-publication history

The pre-publication history for this paper can be accessed here:

http://www.biomedcentral.com/1471-2350/12/133/prepub
